# Evaluation of the bacterial diversity in the feces of cattle using 16S rDNA bacterial tag-encoded FLX amplicon pyrosequencing (bTEFAP)

**DOI:** 10.1186/1471-2180-8-125

**Published:** 2008-07-24

**Authors:** Scot E Dowd, Todd R Callaway, Randall D Wolcott, Yan Sun, Trevor McKeehan, Robert G Hagevoort, Thomas S Edrington

**Affiliations:** 1 USDA-ARS Livestock Issues Research Unit, Lubbock, TX 79403, USA; 2 USDA-ARS, Food and Feed Safety Research Unit, College Station, TX 77845, USA; 3 Medical Biofilm Research Institute, Lubbock, TX 79410, USA; 4 Research and Testing Laboratory, Lubbock, TX 79410, USA; 5 New Mexico State University, Clovis, NM 88101, USA

## Abstract

**Background:**

The microbiota of an animal's intestinal tract plays important roles in the animal's overall health, productivity and well-being. There is still a scarcity of information on the microbial diversity in the gut of livestock species such as cattle. The primary reason for this lack of data relates to the expense of methods needed to generate such data. Here we have utilized a bacterial tag-encoded FLX 16s rDNA amplicon pyrosequencing (bTEFAP) approach that is able to perform diversity analyses of gastrointestinal populations. bTEFAP is relatively inexpensive in terms of both time and labor due to the implementation of a novel tag priming method and an efficient bioinformatics pipeline. We have evaluated the microbiome from the feces of 20 commercial, lactating dairy cows.

**Results:**

Ubiquitous bacteria detected from the cattle feces included *Clostridium*, *Bacteroides, Porpyhyromonas, Ruminococcus, Alistipes, Lachnospiraceae, Prevotella, Lachnospira, Enterococcus, Oscillospira, Cytophage, Anaerotruncus*, and *Acidaminococcus *spp. Foodborne pathogenic bacteria were detected in several of the cattle, a total of 4 cows were found to be positive for *Salmonella *spp (tentative *enterica*) and 6 cows were positive for *Campylobacter *spp. (tentative *lanienae*).

**Conclusion:**

Using bTEFAP we have examined the microbiota in the feces of cattle. As these methods continue to mature we will better understand the ecology of the major populations of bacteria the lower intestinal tract. This in turn will allow for a better understanding of ways in which the intestinal microbiome contributes to animal health, productivity and wellbeing.

## Background

Research on the microbial diversity of the gastrointestinal of livestock is surprisingly scarce, even though it is well understood that bacteria in the gut are vital components that contribute to an animals' health and well-being. The bacterial populations that reside in the gut of animals are diverse and numerous; intestinal populations often exceed 10^11 ^CFU/gram feces [1,2]. The majority of these bacteria are vital to the maintenance of animal's health and it is expected that even minor perturbations in these populations may cause dramatic shifts that can affect livestock productivity [3-5]. These beneficial health effects relate to the ability of these intestinal bacterial populations to supply vital nutrients, convert metabolites and beneficially interact with host cells [6-8]. Information on microbial diversity within the gastrointestinal tract of humans has increased in recent years as a result of 16S rDNA-based analyses [9,10], yet similar data on the microbiomes of livestock is lacking [11,12].

The primary reasons for the lack of knowledge regarding the composition of the intestinal microbiome relates to the difficulty and expense of methods used to evaluate these populations. Traditionally culture-based methods have been used to identify and enumerate commensal members of the ruminal and intestinal flora [13-15]. Culture-based methods are extremely time-consuming and to date we have only been able to culture approximately 1% of the bacteria in the gut [16]. Thus, culture based methods are extremely biased in their evaluation of microbial diversity, tending to overestimate the importance of bacterial species such as *Escherichia coli *that easily grow on an agar surface.

Molecular methodologies developed over the past decade now enable researchers to examine the diversity of the gut microflora independent of cultural methods [17]. Although molecular approaches can also introduce their own forms of bias such as the ability to detect both viable and non-viable bacteria [18-20] they currently provide the most powerful tools available for elucidating the diversity of animal microbiomes [4,11,12,15]. The use of rapid sequencing technologies combined with molecular methods is becoming a gold standard for evaluating the microbiomes of animals [21-25]. In the present study we utilized a novel tag bacterial diversity amplification method that uses massively parallel pyrosequencing techniques to determine the diversity within the intestinal microbiota.

## Results and Discussion

Recent research related to host physiology (obesity) and the gastrointestinal bacterial populations have sparked a renewed interest in understanding the gut microbiome [26,27]. Further studies have indicated that, in humans, intestinal microbial populations of clostridia could be responsible for the development of autism [28,29]. These studies gained much attention because they have correlated physiological conditions with specific microbial populations in the gut. Such studies have raised a pertinent question: is there a microbiome profile in food animals that can increase production efficiency, product quality, and/or food safety? This question has been obliquely addressed through the use of probiotics, prebiotics and competitive exclusion products which seek to establish a healthy "normal" gastrointestinal flora in animals that can improve animal performance or prevent colonization of the animal with pathogens, including zoonotic pathogens [3,30,31].

In our present study, the bTEFAP analysis of fecal samples from 20 individual dairy cows displayed a high diversity of bacterial species and genera. Among these cows there were 274 different bacterial species detected corresponding to 142 separate genera. As several thousand sequences per sample were analyzed (minimum 1732, maximum 3224) we were able to detect populations below 0.1 %, giving a relatively deep and thorough examination of the predominant bacterial populations in these fecal samples.

It has been indicated that the microbial population of lower intestinal bacteria of cattle are dominated by strict anaerobes such as *Bacteroides *spp., *Clostridium *spp., and *Bifidobacterium *spp while facultative anaerobes, such as the enterobacteriaceae (e.g. *E. coli*), are typically reported to occur in numbers at least 100-fold lower than the strict anaerobes [32]. This supports findings from the current study in which the predominant genera found in each of the samples were *Clostridium*, *Bacteroides, Porphyromonas, Ruminococcus, Alistipes, Lachnospiraceae, Prevotella, Lachnospira, Bacteroidales, Akkermansia*, and *Enterococcus *spp (Table 1). We can see that each of the dairy fecal samples was surprisingly consistent in that *Clostridium*, *Porphyromonas*, *Bacteroides*, *Ruminococcus*, *Alistipes*, *Lachnospira*, and *Prevotella *spp were highly prevalent and found in all of the cattle samples.

**Table 1:** Most ubiquitous genera identified from the cow fecal samples (n = 20 cows).

ID	Number of sequences of each genus	Number of cow samples containing each genus	Average % of population across all cows (std dev)	Range of population from all cows (%)
*Clostridium *spp	8701	20	19.0 (3.57)	13.9–25.4
*Bacteroides *spp	4326	20	9.26 (2.17)	5.2–13.7
*Porphyromonas *spp	3435	20	7.34 (2.28)	2.08–11.7
*Ruminococcus *spp	3286	20	3.57 (1.5)	0.79–6.96
*Alistipes *spp	3051	20	6.61 (1.35)	3.54–8.71
Lachnospiraceae-like	2716	20	5.7 (2.77)	2.31–12.2
*Prevotella *spp	2499	20	5.47 (2.13)	2.31–9.89
*Porphyromonas*-like	2097	14	6.37 (2.02)	0.61–11.21
*Bacteroidales *spp	1871	20	4.11 (2.36)	1.1–9.9
*Lachnospira *spp	1753	20	3.73 (2.18)	0.5–7.1
*Akkermansia *spp	1464	19	3.42 (1.97)	0.56–8.64
*Enterococcus *spp	1335	20	2.95 (1.91)	0.73–7.89
*Firmicutes *spp	883	20	1.88 (0.88)	0.36–3.9
*Oscillospira *spp	751	20	1.59 (0.62)	0.2–2.48
Prevotellaceae-like	747	13	2.6 (3.19)	0.1–11.03
Cytophaga spp	638	20	1.35 (0.76)	0.15–2.95
*Eubacterium *spp	598	19	1.31 (0.53)	0.47–2.74
*Francisella *spp	575	15	1.65 (0.75)	0.16–1.65
*Clostridiales *spp	534	20	1.15 (0.58)	0.47–2.51
*Papillibacter *spp	498	18	1.13 (0.75)	0.26–2.41
*Spiroplasma *spp	490	19	1.13 (0.52)	0.39–2.37
*Sedimentibacter *spp	411	18	1.04 (0.77)	0.39–3.74
*Treponema *spp	409	19	0.93 (0.54)	0.12–1.7
*Victivallis *spp	371	13	1.14 (0.86)	0.27–3.19
*Peptococcus *spp	310	19	0.71 (0.49)	0.16–1.94
*Escherichia *spp	254	17	0.68 (0.75)	0.11–3.11
*Anaerotruncus *spp	245	20	0.54 (0.24)	0.19–1.01
*Anaerophaga *spp	216	10	0.9 (0.44)	0.41–1.83
*Acidaminococcus *spp	206	20	0.46 (0.23)	0.15–1.16
*Paenibacillus *spp	194	13	0.59 (0.29)	0.13–1.15
*Streptococcus *spp	193	15	0.55 (0.31)	0.17–1.16
*Fucophilus *spp	191	15	0.53 (0.26)	0.17–1.03
*Flavobacteriaceae *spp	191	11	0.81 (0.94)	0.19–3.43
*Alterococcus *spp	190	10	0.78 (0.39)	0.26–1.53
*Chryseobacterium *spp	187	15	0.53 (0.29)	0.13–1.02
*Catabacter *spp	169	11	0.64 (0.42)	0.16–1.29
Unknown-clusterC	168	13	0.56 (0.41)	0.13–1.33
*Peptostreptococcus *spp	149	15	0.44 (0.30)	0.1–1.17
*Roseburia *spp	146	11	0.59 (0.41)	0.14–1.59
*Sporobacter *spp	141	15	0.41 (0.29)	0.11–1.31
Clostridiaceae-like	117	11	0.45 (0.24)	0.16–0.87
*Acholeplasma *spp	94	11	0.37 (0.25)	0.12–0.88
Unknown-clusterP	65	10	0.29 (0.15)	0.12–0.55

Complete data is provided in supplemental data files. The ID is the genera identified ordered by most abundant sequences. The number of cows that were positive for each genus, the average percentage of the total bacterial population across all cows, and the range of the total population represented by each genus across all cows sampled is also shown in the table.

*Clostridium *spp. is a broad genus and has been described as a "trash can" genus (Steve Zinder, Cornell University, Personal communication), and are ubiquitous in the gastrointestinal tract. Clostridia can both positively and negatively influence the host animal. These effects are typically specifically associated with the individual *Clostridium *species involved [33,34]. Many have negative influences on animal health including species such as *C. perfringens*, *C*. *tetani, C. botulinum*, and *C. difficile *[35-37] and can also cause significant productivity problems including reducing the protein availability in fresh forage diets [38]. Conversely, some *Clostridium *spp. may also be beneficial and improve digestion of complex organic matter such as cellulose and even act as beneficial probiotics [39-43]. In the present study, we detected total of 37 separate species of *Clostridium *spp. (tentatively *straminisolvens, hathewayi, leptum, fimetarium, orbiscindens, lactatifermentans *were the most prevalent) and *Clostridium *spp. was the most common and diverse genus identified. *Clostridium *spp. accounted for approximately 20% of the total microbial populations and were detected in all of the cattle in this study.

*Bacteroides *spp. were also identified in all 20 cattle (and tentatively represented the species *stercoris, denticanum, vulgates, caccae, cillulosolvens*). Bacteroides are well-known intestinal bacteria that can be both beneficial and harmful [44]. Bacteroides are also noted to participate in natural genetic transfer of antimicrobial resistance genes [45]. Another genus that was highly prevalent in the feces of these dairy cattle was *Porphyromonas *spp. There was no clear identification for the most prevalent *Porphyromonas *species in this study though *cangingivalis*, and *levii *were two of the tentative species identified; *P*. *levii *has been associated with bovine necrotic vulvovaginitis [46] and bovine footrot [47]. Little else is known about the role of this bacteria in the gut though one other study identified this bacteria as part of the intestinal community of chickens [48]. Thus it appears that this bovine pathogen may have a reservoir in the feces so that it can be spread to the vulva and feet where it causes disease in cattle.

*Alistipes *spp (tentative *finegoldii*) and *Prevotella *spp. were previously classified as members of the genus *Bacteroides *[49,50]. Other than this original description there are only a handful of reports of *Alistipes *as a member of the intestinal population, including one study which identified this organism as being isolated from the ceca of turkeys [51]. *Prevotella *spp (tentative *oralis*, *ruminicola *and *albensis*) is a well known genus associated with the rumen of cattle [49,52-54] and is associated with ruminal carbohydrate and protein fermentation. *Lachnospira *spp. (tentative *pectinoschiza*) is another genus which has been poorly characterized in environments other than the rumen and has only been occasionally detected in the feces of pigs and humans [55,56]. This however may be one of the first studies to show these genera as a predominant population in the lower intestinal tract of cattle.

Generic *E*. *coli *are easily cultured and ubiquitous in the feces of animals so that they are often used as a marker of fecal contamination in water supplies, however they typically comprise less than 1% of the intestinal bacterial populations [32]. The colony forming unit counts of *E coli *in feces are typically in the 10^4 ^to 10^6 ^range while total microbial counts are in the 10^10 ^to 10^11 ^bacteria per gram of feces range[32]. Because of this it is not surprising that *E*. *coli *were not detected in feces from three of the cows. These results are reflective of the culture-based bias inherent to studies enumerating the easily grown *E*. *coli in vitro *while major populations such as *Clostridium *and *Bacteroides *spp. are fastidious and typically require specialized anaerobic growth conditions.

Zoonotic pathogenic bacteria, such as *Salmonella enterica *and *E*. *coli *O157:H7 can live in the lower gut of cattle and cause human illnesses through carcass contamination, farm run-off, or crop contamination [57-60]. Many live animal anti-pathogen interventions that are currently marketed or have been proposed share a mode of action that alters the microbial ecology of the gut to exclude or to push out these pathogens [61,62]. However, in order to effectively utilize beneficial microbial populations against foodborne pathogens we must understand the normal ecology of the gastrointestinal tract. There are an estimated 1.4 million illnesses and over 500 deaths attributed to salmonellosis in the United States annually [63]. *Salmonella enterica *is a common inhabitant of the gastrointestinal tracts of cattle. Consequently beef and dairy products are also well known sources of human Salmonellosis [59,64-66]. In the present study using bTEFAP, we detected *Salmonella *spp. in 4 of the cattle fecal samples. Similarly, *Campylobacter *is another major cause of foodborne illness [63] and was detected in 6 of the cow samples (Additional file 1). The zoonotic pathogen *E. coli *serotype O157:H7 is commonly associated with the intestinal mucosa of cattle [67]. Although the bTEFAP analysis method has been shown to have the ability to differentiate this serotype within *E*. *coli *(data not shown) they were not detected or differentiated in this study. From the perspective of rapid pathogen detection, one of the most interesting observations from this study was the ability of bTEFAP to detect *Salmonella *spp and *Campylobacter *spp (Supplemental data). This finding illustrates the potential use of the bTEFAP technology as a universal bacterial diagnostic and screening tool for bacterial pathogens and indicates the potential power of bTEFAP as a screening tool in epidemiological studies in animals and humans.

Most of the existing studies seeking to evaluate microbial diversity in the intestinal tract utilize fingerprinting methodologies such as denaturing gradient gel electrophoresis (DGGE) [68]. Fingerprinting methods typically ignore identities of the microbial populations in favor of a simple but important measure of diversity. Culture-based methods that have been used in other diversity estimates and of course over-represent the genera that can be grown easily *in vitro *[69,70]. A number of studies [11] have also evaluated powerful yet classical sequencing approaches, which involve PCR amplification, cloning and Sanger sequencing. Even accounting for potential bias of molecular methods, it is apparent that such methods are the most powerful tools currently available for evaluating the intestinal microbial population of animals. Widespread use of molecular methodologies may usher in a new age in which such diversity studies are no longer limited to a handful of laboratories with abundance of funding and labor.

## Conclusion

In the field of animal nutrition, the microbial processes within the rumen and intestinal tract of food animals remain largely a "black box". Investigators such as Robert Hungate and Marvin Bryant characterized some ruminal and intestinal microbial populations, and related the biochemistry of these microorganisms to their roles in animal nutrition [1,71]; however they were limited to the use of culture-based methodologies. The new method of bTEFAP is not limited to detecting organisms via culture methods, and can be used to define what constitutes a "healthy" or an "unhealthy" microbiome profile by correlating populations of bacterial species with dietary energy and protein utilization, host growth rate and efficiency, host gene expression, and host immune function [72-75]. Recent research aimed at humans has underscored the role that intestinal microbial populations plays in human health and physiology. Thus fully understanding the diversity of the gut community in animals, and how these populations and communities relate to animal performance and displacement of zoonotic pathogen infections in livestock is crucial to making improvements in animal health, productivity and food safety. As this bTEFAP method matures we should have a vastly improved ability to evaluate and monitor changes in microbiomes such as those in the gastrointestinal tract of livestock.

## Methods

### Cattle samples

Fecal grab samples were collected from adult, lactating Holstein dairy cattle (n = 20) on a large (> 3,000 head herd) in the Southwestern United States. Cattle were fed a typical Total Mixed Ration (TMR) commonly fed to dairy cattle in the southwestern U.S. The ration was comprised of chopped alfalfa hay (approximately 20% DM of total ration) and a mixture of cracked corn, soybean meal, cottonseed meal and trace mineral salts. The cows ranged from 18 to 217 d of lactation. Cows were visibly healthy and no illnesses amongst these cows were reported subsequent to the collection. Samples (30–50 g each) were each collected from freshly voided pats off the pen floor. Fecal samples were stored on wet ice and shipped overnight to the laboratory for analysis.

### DNA extraction

Total genomic DNA was extracted from fecal samples using a QIAamp stool DNA mini kit and its manufacturers recommended methods (Qiagen, Valencia, CA). DNA samples were quantified using a Nanodrop spectrophotometer (Nyxor Biotech, Paris, France).

#### bTEFAP Sequencing PCR

The bTEFAP method was performed by the Research and Testing Laboratory (Lubbock, TX). All DNA samples were adjusted to 100 ng/μl. A 100 ng (1 μl) aliquot of each samples DNA was used for a 50 μl PCR reaction. The 16S universal Eubacterial primers 530F (5'-GTG CCA GCM GCN GCG G) and 1100R (5'-GGG TTN CGN TCG TTG) were used for amplifying the 600 bp region of 16S rRNA genes. HotStarTaq Plus Master Mix Kit (Qiagen, Valencia, CA) was used for PCR under the following conditions: 94°C for 3 minutes followed by 32 cycles of 94°C for 30 seconds; 60°C for 40 seconds and 72°C for 1 minute; and a final elongation step at 72°C for 5 minutes. A secondary PCR was performed for FLX (Roche, Nutley, New Jersey) amplicon sequencing under the same condition by using designed special fusion primers with different tag sequences as: LinkerA-Tags-530F and LinkerB-1100R (Table 2). The use of a secondary PCR prevents amplification any potential bias that might be caused by inclusion of tag and linkers during initial template amplification reactions. After secondary PCR all amplicon products from different samples were mixed in equal volumes, and purified using Agencourt Ampure beads (Agencourt Bioscience Corporation, MA, USA). As a note: mixing of the reactions based upon amplicon concentrations rather than volume is preferred.

**Table 2:** Primer sequences utilized for pig samples during bTEFAP

Name	Primer sequence (5'-3')
454-F30	GCCTCCCTCGCGCCATCAGCGCACTACGTGTGCCAGCMGCNGCGG
454-F31	GCCTCCCTCGCGCCATCAGCGCAGCTGTTGTGCCAGCMGCNGCGG
454-F32	GCCTCCCTCGCGCCATCAGCGCATACAGTGTGCCAGCMGCNGCGG
454-F33	GCCTCCCTCGCGCCATCAGCGCATCTATAGTGCCAGCMGCNGCGG
454-F34	GCCTCCCTCGCGCCATCAGCGCATTGGTGGTGCCAGCMGCNGCGG
454-F35	GCCTCCCTCGCGCCATCAGCGCCAGAAAAGTGCCAGCMGCNGCGG
454-F36	GCCTCCCTCGCGCCATCAGTGTGACGTACGTGCCAGCMGCNGCGG
454-F37	GCCTCCCTCGCGCCATCAGTGTGTGCATAGTGCCAGCMGCNGCGG
454-F38	GCCTCCCTCGCGCCATCAGTGTGTCCTCAGTGCCAGCMGCNGCGG
454-F39	GCCTCCCTCGCGCCATCAGTGTGCATCACGTGCCAGCMGCNGCGG
454-F40	GCCTCCCTCGCGCCATCAGTGTGCCTAGAGTGCCAGCMGCNGCGG
454-F41	GCCTCCCTCGCGCCATCAGTGTACATAGTGTGCCAGCMGCNGCGG
454-F42	GCCTCCCTCGCGCCATCAGTGTACATTGAGTGCCAGCMGCNGCGG
454-F43	GCCTCCCTCGCGCCATCAGTGTACATTGTGTGCCAGCMGCNGCGG
454-F44	GCCTCCCTCGCGCCATCAGTGTACCAACAGTGCCAGCMGCNGCGG
454-F45	GCCTCCCTCGCGCCATCAGTGTACCAACTGTGCCAGCMGCNGCGG
454-F46	GCCTCCCTCGCGCCATCAGTGTACCAATCGTGCCAGCMGCNGCGG
454-F47	GCCTCCCTCGCGCCATCAGTGTACCAGATGTGCCAGCMGCNGCGG
454-F48	GCCTCCCTCGCGCCATCAGTGTACCCATAGTGCCAGCMGCNGCGG
454-F49	GCCTCCCTCGCGCCATCAGTGTACAGGGTGTGCCAGCMGCNGCGG
454-F50	GCCTCCCTCGCGCCATCAGTGTACCTATCGTGCCAGCMGCNGCGG
linkerB-1100R	GCCTTGCCAGCCCGCTCAGGGGTTNCGNTCGTTG

#### bTEFAP FLX massively parallel pyrosequencing

In preparation for FLX sequencing (Roche, Nutley, New Jersey), the DNA fragments size and concentration were accurately measured by using DNA chips under a Bio-Rad Experion Automated Electrophoresis Station (Bio-Rad Laboratories, CA, USA) and a TBS-380 Fluorometer (Turner Biosystems, CA, USA). A 9.6 E+06 sample of double-stranded DNA molecules/μl with an average size of 625 bp were combined with 9.6 million DNA capture beads, and then amplified by emulsion PCR. After bead recovery and bead enrichment, the bead-attached DNAs were denatured with NaOH, and sequencing primers were annealed. A two-region 454 sequencing run was performed on a 70×75 GS PicoTiterPlate (PTP) by using a Genome Sequencer FLX System (Roche, Nutley, New Jersey). It should be noted that 100 total samples were run within this same FLX 2-region sequencing reaction. The additional 79 tagged samples were associated with unrelated studies. All FLX related procedures were performed following Genome Sequencer FLX System manufacturers instructions (Roche, Nutley, New Jersey).

#### bTEFAP Tag design

A custom script written in C# was utilized to generate all possible combinations of 10-mer oligonucleotide tags with GC % between 40 and 60%. From this pool we then chose 20 individual tags (Table 2).

#### bTEFAP Sequence processing pipeline

Custom software written in C# within a Microsoft^® ^.NET (Microsoft Corp, Seattle, WA) development environment was utilized for all post sequencing processing. Discussion of software code is outside the scope of this report however a description of the algorithm follows. Quality trimmed sequences obtained from the FLX sequencing run were processed using a custom scripted bioinformatics pipeline. In short, quality trimmed sequencing reads were derived directly from FLX sequencing run output files. Tags were extracted from the multi-FASTA file into individual sample specific files based upon the tag sequence. Tags which did not have 100% homology to the sample designation were not considered. Sequences which were less than 150 bp after quality trimming were not considered. The total number of sequences among the 20 samples was 49635. These sequences were fairly divided among the 20 samples averaging close to 2500 sequence reads per sample. The resultant individual sample after parsing the tags into individual FASTA files were assembled using CAP3 [76]. The ace files generated by CAP3 were then processed to generate a secondary FASTA file containing the tentative consensus (TC) sequences of the assembly along with the number of reads integrated into each consensus. TC were required to have at least 3-fold coverage. The resulting TC FASTA for each sample was then evaluated using BLASTn [77] against a custom database derived from the RDP-II database [78] and GenBank . The sequences contained within the curated 16S database were both > 1200 bp and considered of high quality based upon RDP-II standards. A post processing algorithm generated best-hit files with E-values < 10e-114 and bit scores > 400. The identities of all hits were greater than 98%. These parameters, based upon an average TC length of 260 bp have been previously evaluated to enable reliable identification at the genus and species level (data not shown). However, identification at the species level will only be considered putative for the purpose of this pilot study. Following best-hit processing a secondary post-processing algorithm was utilized to combine genus designations generating a list of genera IDs and their relative predicted abundance within the given sample.

#### Statistics

Statistics were performed using the Basic comparative functions of JMP 6.0 (SAS institute, Cary, NC).

## Authors' contributions

SED helped conceived of the project, developed the methods and software, wrote first drafts of the manuscript, TRC helped conceive of the project, drafting of the manuscript, performed sample collection, RDW assisted with the development of methods, YS performed bTEFAP laboratory studies, TM was responsible for software programming, RGH managed animal aspects of the project, TSE helped with animal studies and manuscript drafts.

## Supplementary Material

Additional File 1 Table providing all of the genus identified in this cattle fecal microbiome pilot study. The data is sorted by the number of fecal samples in which each genera was detected and then by the total number of sequences corresponding to this genera. Click here for file
