# Supplementary material for: Evaluation of the bacterial diversity in the feces of cattle using 16S rDNA bacterial tag-encoded FLX amplicon pyrosequencing (bTEFAP)
Source: BMC Microbiol. 2008 Jul 24;8:125. doi: 10.1186/1471-2180-8-125 (PMC2515157; doi:10.1186/1471-2180-8-125)
Supplement: Additional File 1 — Table providing all of the genus identified in this cattle fecal microbiome pilot study. The data is sorted by the number of fecal samples in which each genera was detected and then by the total number of sequences corresponding to this genera. [file 1471-2180-8-125-S1.doc]

| genus | #seq | # samples |
| --- | --- | --- |
| Clostridium spp | 8701 | 20 |
| Bacteroides spp | 4326 | 20 |
| Porphyromonas spp | 3435 | 20 |
| Alistipes spp | 3051 | 20 |
| Lachnospiraceae-like | 2716 | 20 |
| Prevotella spp | 2499 | 20 |
| Rumenococcus spp | 3286 | 20 |
| Bacteroidales spp | 1871 | 20 |
| Lachnospira spp | 1753 | 20 |
| Enterococcus spp | 1335 | 20 |
| Firmicutes spp | 883 | 20 |
| Oscillospira spp | 751 | 20 |
| Cytophaga spp | 638 | 20 |
| Clostridiales spp | 534 | 20 |
| Anaerotruncus spp | 245 | 20 |
| Acidaminococcus spp | 206 | 20 |
| Akkermansia spp | 1464 | 19 |
| Eubacterium spp | 598 | 19 |
| Spiroplasma spp | 490 | 19 |
| Treponema spp | 409 | 19 |
| Peptococcus spp | 310 | 19 |
| Papillibacter spp | 498 | 18 |
| Sedimentibacter spp | 411 | 18 |
| Escherichia spp | 254 | 17 |
| Francisella spp | 575 | 15 |
| Streptococcus spp | 193 | 15 |
| Fucophilus spp | 191 | 15 |
| Chryseobacterium spp | 187 | 15 |
| Peptostreptococcus spp | 149 | 15 |
| Sporobacter spp | 141 | 15 |
| Porphyromonas-like spp | 2097 | 14 |
| Prevotellaceae-like | 747 | 13 |
| Victivallis spp | 371 | 13 |
| Paenibacillus spp | 194 | 13 |
| Unknown-cluster | 168 | 13 |
| Flavobacteriaceae spp | 191 | 11 |
| Catabacter spp | 169 | 11 |
| Roseburia spp | 146 | 11 |
| Clostridiaceae spp | 117 | 11 |
| Acholeplasma spp | 94 | 11 |
| Anaerophaga spp | 216 | 10 |
| Alterococcus spp | 190 | 10 |
| Unknown-potato | 65 | 10 |
| Hespellia spp | 109 | 9 |
| Ethanologenbacterium spp | 107 | 9 |
| Flavobacterium spp | 76 | 9 |
| Turicibacter spp | 74 | 9 |
| Bacillus spp | 52 | 9 |
| Acetanaerobacterium spp | 56 | 8 |
| Succinivibrio spp | 54 | 8 |
| Desulfovibrio spp | 46 | 8 |
| Anaeroplasma spp | 45 | 8 |
| Bacteroidetes spp | 163 | 7 |
| Anaerostipes spp | 63 | 7 |
| Faecalibacterium spp | 59 | 7 |
| Erysipelothrix spp | 51 | 7 |
| Butyrivibrio spp | 35 | 7 |
| Dolosicoccus spp | 56 | 6 |
| Alkalibacter spp | 54 | 6 |
| Campylobacter spp | 43 | 6 |
| Holdemania spp | 39 | 6 |
| Mycoplasma spp | 34 | 6 |
| Selenomonas spp | 26 | 5 |
| Unknown | 65 | 4 |
| Cryomorphaceae spp | 59 | 4 |
| Salmonella spp | 40 | 4 |
| Psychrobacter spp | 40 | 4 |
| Unknown | 33 | 4 |
| Parasporobacterium spp | 31 | 4 |
| Unknown | 23 | 4 |
| Prolixibacter spp | 21 | 4 |
| Pectinatus spp | 18 | 4 |
| Bacteroides-like spp | 226 | 3 |
| Unknown | 35 | 3 |
| Anaerofilum spp | 25 | 3 |
| Desulfotomaculum spp | 25 | 3 |
| Ruminobacter spp | 21 | 3 |
| Coprococcus spp | 21 | 3 |
| Unknown | 20 | 3 |
| Sutterella spp | 19 | 3 |
| Octadecabacter spp | 12 | 3 |
| Rhizobiaceae spp | 109 | 2 |
| Tannerella spp | 90 | 2 |
| Maorithyas spp | 51 | 2 |
| Unknown | 21 | 2 |
| Epixenosomes spp | 17 | 2 |
| Epulopiscium spp | 16 | 2 |
| Acetivibrio spp | 15 | 2 |
| Anaerovibrio spp | 14 | 2 |
| Caryophanon spp | 11 | 2 |
| Napier spp | 11 | 2 |
| Thermodesulforhabdus spp | 10 | 2 |
| Facklamia spp | 10 | 2 |
| Rhodopseudomonas spp | 8 | 2 |
| Parvulomonas spp | 8 | 2 |
| Unknown | 6 | 2 |
| Candidatus spp | 72 | 1 |
| Woodsholea spp | 45 | 1 |
| Flexibacter spp | 45 | 1 |
| Unknown | 30 | 1 |
| Unknown | 16 | 1 |
| Caloramator spp | 15 | 1 |
| Pedobacter spp | 13 | 1 |
| Asteroleplasma spp | 12 | 1 |
| Caulobacter spp | 12 | 1 |
| Mollicutes spp | 9 | 1 |
| Unknown | 9 | 1 |
| Anaerofustis spp | 9 | 1 |
| Unknown | 8 | 1 |
| Olavius spp | 7 | 1 |
| Desulfonema spp | 7 | 1 |
| Filifactor spp | 7 | 1 |
| Ralstonia spp | 6 | 1 |
| Beggiatoa spp | 5 | 1 |
| Rikenella spp | 5 | 1 |
| Rhodovibrio spp | 4 | 1 |
| Smithella spp | 4 | 1 |
| Dyadobacter spp | 4 | 1 |
| Methylobacterium spp | 4 | 1 |
| Vietnam spp | 4 | 1 |
| Leuconostoc spp | 3 | 1 |
| Megamonas spp | 3 | 1 |
| Flexibacteraceae spp | 3 | 1 |
| Cassava spp | 3 | 1 |
| Metabacterium spp | 3 | 1 |
| Rhodococcus spp | 3 | 1 |
| Ophiactis spp | 3 | 1 |
| Sphingomonas spp | 3 | 1 |
| Synechococcus spp | 3 | 1 |
| Unknown | 3 | 1 |
| Dysgonomonas spp | 3 | 1 |
| Acetitomaculum spp | 3 | 1 |
| Chthoniobacter spp | 3 | 1 |
| Oxobacter spp | 3 | 1 |
| Dehalobacterium spp | 3 | 1 |
| Spirochaeta spp | 3 | 1 |
| Mogibacterium spp | 3 | 1 |
| Piscirickettsia spp | 3 | 1 |
| Entomoplasma spp | 3 | 1 |
| Staphylococcus spp | 3 | 1 |
| Eubacteriaceae spp | 3 | 1 |
